# Detecting space–time clusters of COVID-19 in Brazil: mortality, inequality, socioeconomic vulnerability, and the relative risk of the disease in Brazilian municipalities

**DOI:** 10.1007/s10109-020-00344-0

**Published:** 2021-03-08

**Authors:** M. R. Martines, R. V. Ferreira, R. H. Toppa, L. M. Assunção, M. R. Desjardins, E. M. Delmelle

**Affiliations:** 1 Department of Geography, Tourism and Humanities, Research Group: Center for Studies in Landscape Ecology and Conservation, Federal University of São Carlos, Sorocaba, SP Brazil; 2 Department of Geography, Research Group: Center for Studies in Landscape Ecology and Conservation, Federal University of Triângulo Mineiro, Uberaba Campus, State of Minas Gerais Brazil; 3 Department of Environmental Sciences, Research Group: Center for Studies in Landscape Ecology and Conservation, Federal University of São Carlos, Sorocaba, SP Brazil; 4 Faculty of Law, State University of Minas Gerais, Ituiutaba Campus, Brazil; 5 Department of Epidemiology, Spatial Science for Public Health Center, Johns Hopkins Bloomberg School of Public Health, Baltimore, MD 21205 USA; 6 Department of Geography and Earth Sciences, Center for Applied Geographic Information Science, University of North Carolina at Charlotte, Charlotte, NC 28223 USA; 7 Department of Geographical and Historical Studies, University of Eastern Finland, 80101 Joensuu, Finland

**Keywords:** Disease surveillance, COVID-19, Geographic information systems, Relative risk, Space–time statistics, Spatial models, C18, C31, I100, C020

## Abstract

The first case of COVID-19 in South America occurred in Brazil on February 25, 2020. By July 20, 2020, there were 2,118,646 confirmed cases and 80,120 confirmed deaths. To assist with the development of preventive measures and targeted interventions to combat the pandemic in Brazil, we present a geographic study to detect “active” and “emerging” space–time clusters of COVID-19. We document the relationship between relative risk of COVID-19 and mortality, inequality, socioeconomic vulnerability variables. We used the prospective space–time scan statistic to detect daily COVID-19 clusters and examine the relative risk between February 25–June 7, 2020, and February 25–July 20, 2020, in 5570 Brazilian municipalities. We apply a Generalized Linear Model (GLM) to assess whether mortality rate, GINI index, and social inequality are predictors for the relative risk of each cluster. We detected 7 “active” clusters in the first time period, being one in the north, two in the northeast, two in the southeast, one in the south, and one in the capital of Brazil. In the second period, we found 9 clusters with RR > 1 located in all Brazilian regions. The results obtained through the GLM showed that there is a significant positive correlation between the predictor variables in relation to the relative risk of COVID-19. Given the presence of spatial autocorrelation in the GLM residuals, a spatial lag model was conducted that revealed that spatial effects, and both GINI index and mortality rate were strong predictors in the increase in COVID-19 relative risk in Brazil. Our research can be utilized to improve COVID-19 response and planning in all Brazilian states. The results from this study are particularly salient to public health, as they can guide targeted intervention measures, lowering the magnitude and spread of COVID-19. They can also improve resource allocation such as tests and vaccines (when available) by informing key public health officials about the highest risk areas of COVID-19.

## Introduction

Over the past 18 years, zoonotic coronavirus transmissions have been a global health concern. During that period, there were two epidemics: SARS-CoV in 2003 in China, which spread across 30 countries in six continents and resulted in 8098 cases and 774 deaths (9.5%), while the second being Middle East Syndrome Coronavirus (MERS-CoV), which started in the Kingdom of Saudi Arabia in 2012 and spread throughout 27 countries with 2494 laboratory-confirmed cases and 858 related deaths (Al-Tawfiq et al. 2014; WHO 2019; Aly et al. 2017). The third time that a zoonotic coronavirus had crossed species and infected humans was SARS-CoV-2 (Perlman 2020)—also called coronavirus disease 2019 (COVID-19)–but it is the first time a coronavirus outbreak is considered to be a pandemic. The first case of COVID-19 in South America occurred in Brazil on February 25, 2020. The country has a high connection with other countries through airports and shipping ports, especially in cities such as São Paulo and Rio de Janeiro, which facilitates the spread of the disease inland and in coastal regions as well as neighboring countries (Rodriguez-Morales et al. 2020; FIOCRUZ 2020).

By June 7, 2020, there were 691,758 confirmed cases and 36,455 confirmed deaths in Brazil, with a mortality rate of 5.3%. By July 20th, the figures increased to 2,118,646 cases and 80,120 deaths with a mortality rate of 3.8% (Brazil 2020). However, underreporting of deaths and lack of testing were also expected to bias these numbers (Alonso et al. 2020). It is generally acknowledged that the highest proportion of deaths by COVID-19 occur among the elderly; those with the most severe disease were most likely having a history of hypertension, respiratory disease, and cardiovascular disease (Jordan et al. 2020; Du et al. 2020). The risk of death among young adults is smaller than that of older adults, e.g., at most 0.1%–0.2% (Jordan et al. 2020; Kobayashi et al. 2020); however, severe outcomes and deaths have also been reported among children (Deyà-Martínez et al. 2020; Jones et al. 2020). Promislow (2020) reported that COVID-19 mortality rates tended to increase exponentially with age, while males tended to have a higher risk of dying across all ages.

COVID-19 has the potential to affect everyone in society; however, the virus affects specific segments of the population very differently, due to their vulnerability (Smith and Judd 2020). Although Brazil has made significant progress in extending a range of social protection (e.g., universal health care), there remains important social inequalities (Landmann-Szwarcwald and Macinko 2016). The poorest segment of the population is the most vulnerable, especially in the time of a crisis, as it is affected by unemployment, the weakening of social safety nets, and access to health services (Ahmed et al. 2020).

Surveillance of COVID-19 is essential to improve response and planning, such as allocating testing and hospital resources, and mitigating the social vulnerability of the population. An effective public health response to the disease requires the ability to monitor and analyze outbreaks under critical space–time conditions. Space–time analytics are particularly attractive to analyze spatial data with a temporal dimension (Carroll et al. 2014; Delmelle et al. 2011, 2014; Jacquez et al. 2005; Levine 2006, Robertson et al. 2010; Rogerson and Yamada 2008; Paez et al. 2020, Yamada et al. 2009), allowing to estimate the dynamics of infectious diseases. The prospective space–time scan statistic (Kulldorff 1997) is a widely used cluster detection tool in disease surveillance, which can identify areas that are statistically significant hotspots of disease incidence on the most current time period of the analysis (Allévius and Höhle 2019). The statistic determines if the space–time patterns of COVID-19 cases exhibit statistically significant clustering. Cylindrical scanning windows of different spatial and temporal dimensions are computed to systematically scan the study area and time period for more observed than expected disease cases. The prospective version of the scan statistic is slightly different than the retrospective version (Desjardins et al. 2018; Owusu et al. 2019; Whiteman et al. 2019) because it disregards historical clusters that may have previously existed before the most current day of analysis (Kulldorff 2001).

There are many examples illustrating the use of the prospective space–time scan statistic. Chen et al. (2016) designed an online analytical tool for frontline public health workers to prospectively detect ongoing dengue fever in each village of Tainan and Kaohsiung transmission on a weekly basis. Tang et al. (2017) identified seasonal peaks and high-risk periods of measles in Guangxi during 2013–2014 and found patterns of transmission in space and time. Al-Ahmadi et al. (2019) provided an initial assessment for the potential environmental risk factors of MERS-CoV in Saudi Arabia between June 2012 and March 2019, performing spatiotemporal cluster analyses proposed by Kulldorff’s spatial scan statistics on cases reported in that period. It was the first study that aims to analyze the spatiotemporal pattern and clustering of MERS-CoV in Saudi Arabia, and the results reinforce that secondary infections are the great challenge for health-care system in the prevention and control of MERS-CoV outbreaks in Saudi Arabia. The prospective scan statistic has recently been used in a series of studies on COVID-19. Desjardins et al. (2020), Hohl et al. (2020a) and Hohl et al. (2020b) identified COVID-19 clusters and estimated relative risk throughout the USA at the county level. Masrur et al. (2020) conducted spatiotemporal analysis using the prospective scanning statistic in Bangladesh, suggesting that the country had experienced a community-level transmission as early as March 2020. Alkhamis et al. (2020) and Gomes et al. (2020) used the same approach to identify clustering events that were still active (i.e., emerging clusters) in the State of Kuwait and Northern Brazil, respectively.

The results from these studies are particularly salient to public health, as they can guide targeted intervention measures, lowering the magnitude and transmission of COVID-19. They can also improve resource allocation and justify continued social distancing and stay-at-home orders by informing key public health officials about the highest risk areas of COVID-19. The importance of the prospective approach is that it can be extended to analyze the characteristics of the population of municipalities within the clusters. As case data are updated, the analysis can be repeated to continuously monitor the evolution of COVID-19 outbreaks (Desjardins et al. 2020).

In Brazil, the State Health Secretariats (SHS) update the data daily and make them public, so our approach is well suited to facilitate daily COVID-19 surveillance in the country. The Ministry of Health reports daily confirmed cases and deaths; while also utilizing a COVID-19 app to disseminate information (de Oliveira et al. 2020). Regarding COVID-19 surveillance in Brazil, a susceptible, exposed, infected, removed (SEIR) model was applied to several lockdown scenarios (Tarrataca et al. 2020); while Ribeiro and Bernardes (2020) estimated the number of underreported cases and deaths in Brazil. Some studies were conducted in Brazil about the risk of COVID-19 transmission and health-care system capacity. Costa et al. (2020) used demographic and mobility data and COVID-19 cases along 3 weeks since March 31st and performed a long-term analysis of epidemic outcomes using a stochastic metapopulation model. The authors found that the degree of heterogeneity and desynchronization of the epidemic curves in cities with large populations and countryside regions suggest diverse mitigation scenarios and strategies to combat COVID-19. Castro et al. (2020) simulated the time it would take for hospitals to operate beyond their capacity in Brazil. According to the onset and the intensity of transmission, shortages of hospital beds, intensive care unit (ICU) beds, and ventilators could affect populations that depend on public health systems, and this highlights issues with equity and ethics in service allocation. Considering cases of COVID-19 in March 2020, Coelho et al. (2020) calculated the probability of COVID-19 spread from São Paulo and Rio de Janeiro, considering human mobility. In addition, they evaluated socioeconomic indices to identify vulnerable areas and concluded that North and Northeast Brazil are high risk and vulnerable to adverse health outcomes.

Utilizing a prospective space–time scan statistic, our objective is to detect new emerging clusters of COVID-19 across 5570 Brazilian municipalities, contrasting two temporal intervals, from February 25 to June 7, 2020, and from February 25 to July 20, 2020. When examining these time periods, we compute the evolution of the relative risk of the clusters in different regions and municipalities in Brazil and find associations with mortality rate, vulnerability, and social inequality.

## Data and methods

### COVID-19 data and geographic information

Brazil is comprised of 5570 municipalities in 26 states. With a population of approximately 210,147,125 people (IBGE 2019), Brazil is the sixth largest country in population and fifth in landmass, which faces great inequalities and socioeconomic disparities. East coast states include approximately 70% of the population. The states of São Paulo and Rio de Janeiro have the highest population density (similar to Europe) while the states of the Amazon region have densities close to those of Canada and Australia (Somain 2014).

**Fig. 1:**
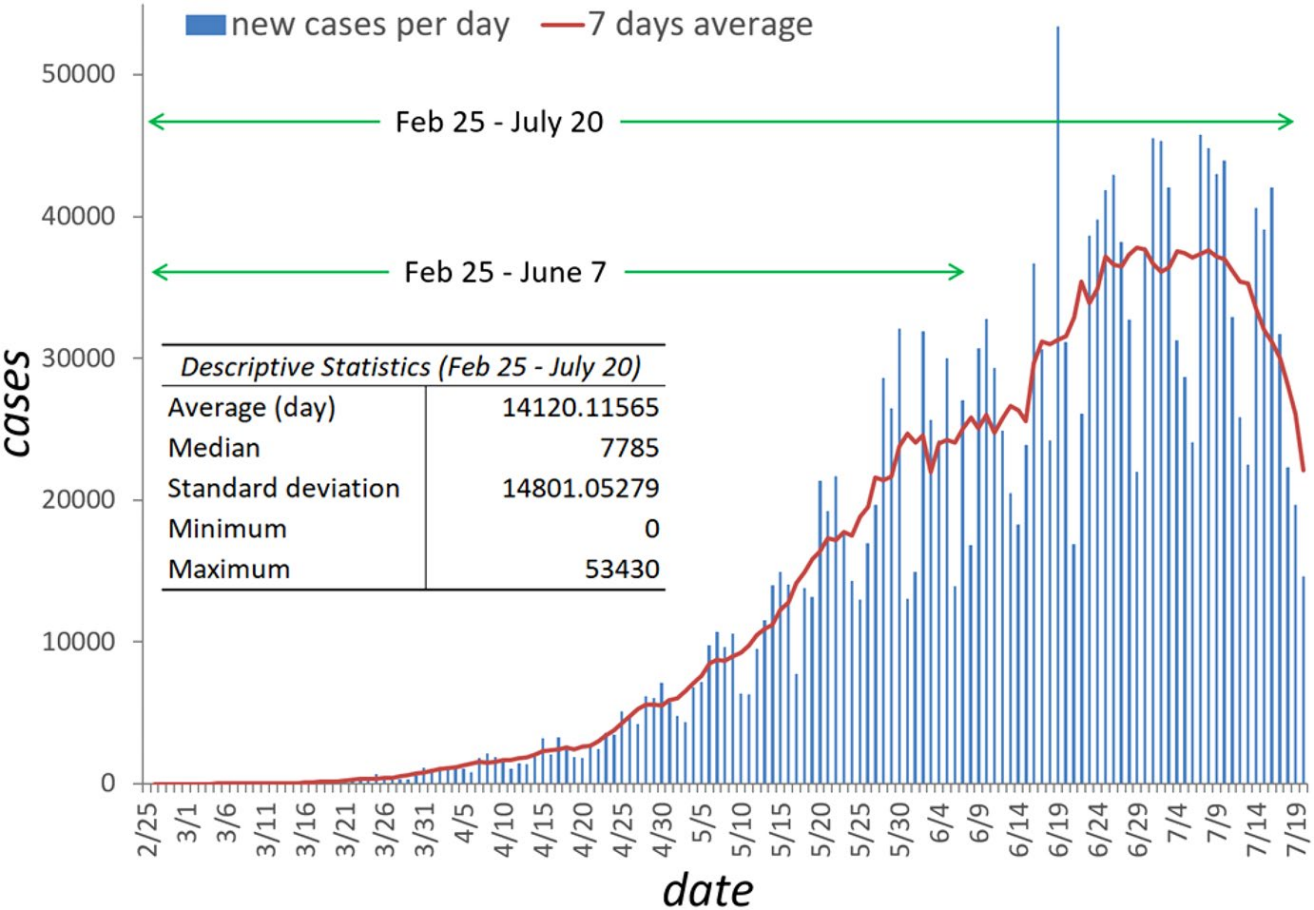
Daily number of reported COVID-19 cases in Brazil between February 25 and June 7, 2020, and descriptive statistics

COVID-19 cases were retrieved from the Brazil.io project website (Brazil IO, 2020). This project compiles data from the daily COVID-19 case reports by municipality in the 27 units of Brazil and are available in a raw format, which were then tabulated to a format SaTScan could support. The data are from February 25, 2020, to July 20, 2020. In Brazil, 2,075,657 cases of COVID-19 were confirmed between the two aforementioned time periods (Fig. 1).

In GIS, two layers of geographic data were utilized: (1) the location of 5570 municipal seats for the SaTScan clusters detection and (2) municipality polygons for visualizing clustering and relative risk results via choropleth maps. These layers were retrieved by the Instituto Brasileiro de Geografia e Estatística (IBGE) website; English—The Brazilian Institute of Geography and Statistics. Using the location of the municipal seat instead of the centroid of the municipality better reflects the population concentration. The data sources are shown in appendix Table 11.

### Space–time cluster detection

The detection of active clusters is conducted using the prospective Poisson space–time scan statistic method (Kulldorff 2001). The statistic detects active clusters of disease on the most current day of analysis (Jones et al. 2006). New data can be added to monitor active and emerging clusters and identify areas that no longer are experiencing excess incidence (e.g., less observed than expected cases). The statistic systematically implements moving cylinders to scan the study area, which are centered on the centroid of the Brazilian municipalities. The base of the cylinder is the spatial scanning window, and the height represents the temporal scanning window, which are both expanded until a maximum threshold is reached. The null hypothesis stipulates that the model reflects an inhomogeneous Poisson process with an intensity μ, which is proportional to the at-risk population. The alternative hypothesis is that the number of reported cases exceeds the number of expected cases derived from the null model. A maximum likelihood ratio test is utilized to evaluate the null and alternative hypotheses, which is defined in Eq. 1:

1 $$\frac{L\left( Z \right)}{{L_{0} }} = \frac{{\left( {\frac{{n_{Z} }}{\mu \left( Z \right)}} \right)^{{n_{Z} }} \left( {\frac{{N - n_{Z} }}{{N - \mu_{\left( Z \right)} }}} \right)^{{N - n_{Z} }} }}{{\left( {\frac{N}{\mu \left( A \right)}} \right)^{N} }}$$

where L(Z) is the likelihood function for cylinder and Ze*L*_0_ is the likelihood function for the null hypotheses for cylinder Z. Essentially, the number of observed disease cases in a cylinder $n_{Z}$ is divided by the number of expected cases in a cylinder $\mu \left( Z \right)$ to the power of the observed $n_{Z}$, multiplied by the observed cases divided by the expected cases outside of the cylinder. The numerator is then divided by the quotient of dividing the total number of observed cases for the entire study area N across all time periods $\mu \left( A \right)$, to the power of the total number of observed cases. The cylinder will have an elevated risk if the likelihood ratio is greater than 1 (i.e., $\frac{{n_{Z} }}{\mu \left( Z \right)} > \frac{{N - n_{Z} }}{{N - \mu_{\left( Z \right)} }}$). Furthermore, the cylinder with the highest likelihood ratio value is the most likely cluster.

The majority of the literature pertaining to STSS only report the locations that belong to a significant space–time cluster. However, this approach assumes that the risk of infection is homogenous throughout the cluster. Conversely, some locations within a cluster may contain zero cases of a particular disease, due to the scanning nature of the STSS. To reduce uncertainty by identifying the municipalities that are the highest risk locations in a cluster (rather than assuming the risk of disease is homogenous throughout a cluster), we also report the relative risk of each areal unit belonging to a space–time cluster, which can provide additional evidence for targeted interventions. Relative risk quantifies the risk of becoming infected with a disease in one location compared to all other locations (Eq. 2):

2 $$RR = \frac{{c/{\text{E}}\left[ {\text{c}} \right]}}{{\left( {C - c} \right)/\left( {C - {\text{E}}\left[ {\text{c}} \right]} \right)}}$$

We defined the maximum spatial and temporal search windows to 10% of the population at-risk and 50% of the study period, respectively. Each cluster’s duration is set to a minimum of 2 days and a cluster must contain a minimum of five confirmed cases of COVID-19 (Desjardins et al. 2020). We utilize a prospective Poisson model to detect space–time clusters that are still occurring or active on June 20, 2020, and July 20, 2020 (Kulldorff 2001; Desjardins et al. 2020). We assume that COVID-19 cases follow a Poisson distribution under the null hypothesis that states that the model reflects a constant risk. The alternative hypothesis states that the number of observed cases exceed the number of expected cases derived from the null model. The expected cases are estimated by multiplying the population in the cylinder by the total COVID-19 rate in each cylinder. A maximum likelihood ratio test is implemented to evaluate whether cylinders have an elevated risk of COVID-19. If the cylinder has a likelihood ratio > 1, then it has an elevated risk—(i.e., case rate within the cylinder is greater than the case rate outside of the cylinder, that is, all municipalities in Brazil). To derive statistical significance, 999 Monte Carlo simulations are computed for each cylinder. We report clusters at the *p* < 0.05 level and map the relative risk of each municipality. The relative risk is defined as the estimated risk of COVID-19 within a municipality divided by the risk outside of the municipality. We utilize the SaTScan software for space–time cluster detection of COVID-19 data and a commercial GIS software for the visualization of clusters and relative risk of the Brazilian municipalities.

### Non-spatial modeling

To examine the role of socioeconomic characteristics on the presence of COVID-19 clusters, we select three indicators reflecting population characteristics and COVID-19 mortality: the GINI index, (IPEA 2015) the Brazilian Social Vulnerability Index (SVI) (Atlas Brasil 2013), and COVID-19 mortality rate. The GINI coefficient has been applied in the area of health to measure disparities (Han et al. 2016) and is based on population income per municipality and ranges between 0 in the case of perfect equality and 1 in the case of perfect inequality. The SVI is an index that varies between 0 and 1 and summarizes three attributes: urban infrastructure, human capital, and income and labor. The closer to 1, the greater the social vulnerability of a municipality. These dimensions correspond to sets of variables that indicate that the standard of living of families is low, suggesting non-access and non-observance of social variables. For municipalities with an SVI between 0 and 0.200, this indicates very low social vulnerability; between 0.201 and 0.300 indicates low social vulnerability; between 0.301 and 0.400 indicates middle social vulnerability; between 0.401 and 0.500 indicates high social vulnerability; and between 0.501 and 1 indicates that the municipality has very high social vulnerability (Brazil 2015). The mortality rate was selected because it is a criticality indicator since it is influenced by the structure of the population, sex, and age, in turn, conditioned by socioeconomic factors.

To analyze the correlation between the RR and the selected independent variables, we used the RR value of each municipality located in the space–time clusters, from February 25, 2020, to July 20, 2020 (*n* = 3304). We evaluate the effect of socioeconomic variables and mortality rate on RR using a Generalized Linear Model (GLM) (Eq 3).

3 $$Y = \beta_{0} + \beta_{1a} + \beta_{2b} + \beta_{3c} + \varepsilon$$

with *Y* the relative risk, *β* the regression coefficients, “a” reflecting the GINI variable, “b” the SVI variable, “c” the mortality rate, and ε the error of the terms. Descriptive statistics for the variables used in the GLM model are provided in appendix Table 12. The GLM technique is conducted in the R software (version 4.0.1.).

### Spatial modeling

We implement a Moran’s I test on the GLM residuals to detect the presence of spatial autocorrelation (Anselin 1988; Anselin and Bera 1998) and justify the use of the subsequent spatial modeling. First, we conduct a Spatial Lag Model (Eq. 4) to estimate how the dependent variable *Y* at in a municipality *i* is affected by its neighboring municipalities *j*.

4 $$Y = \beta_{0} + \lambda WY + X\beta + \varepsilon$$

where *Y* denotes the vector of the response variables, *Xβ* is the dimensionality of the vector parameter for the variables considered (GINI values, SVI values and mortality rate), *λ* is the autoregressive spatial coefficient (when *λ* = 0 the autocorrelation is null), *WY* expresses the spatial dependence *Y*. Second, we test the Spatial Error Model (Eq. 5), which controls the spatial autocorrelation in the residuals, and thus in both dependent and independent variables.

5 $$Y = \beta_{0} + \, X\beta + \rho W\varepsilon + \xi$$

where *Wε* is the error with spatial effect and ρ*Wε* is the measure of the autocorrelation of the errors of *Y*, and *ξ* the error component with constant and uncorrelated variance (white noise).

We applied the Lagrange Multiplier (LM) test to identify the model with the strongest explanatory power for the variable *Y* (Anselin 2005). This test estimates the LM-Lag for the dependency in relation to the original variables in the neighboring areas and the LM-Error in relation to the residuals in the neighboring areas. If significant, this indicates that a spatial regression may capture some of the spatial effects that affect the behavior of the *Y* variable; if both models are significant, the best model should be selected according to the Akaike Information Criterion (AIC) (Anselin 1996, 1988; Anselin and Bera 1998). We used the Jarque–Bera test to examine the normality of the distribution of the errors (Anselin et al. 2010).

When a spatial model is utilized, it is recommended to check whether the dependent variable of the target location is influenced by neighboring locations (Lesage and Pace 2009). In this context, it is possible to identify spillover effects among neighboring municipalities. For example, evaluating whether the COVID-19 relative risk of a municipality is positively or negatively related to the RR of neighboring municipalities. Here, we use GeoDA (Anselin 2005) to compute the spatial regression models.

## Results

### Emerging clusters, February 25–June 7, 2020

**Fig. 2:**
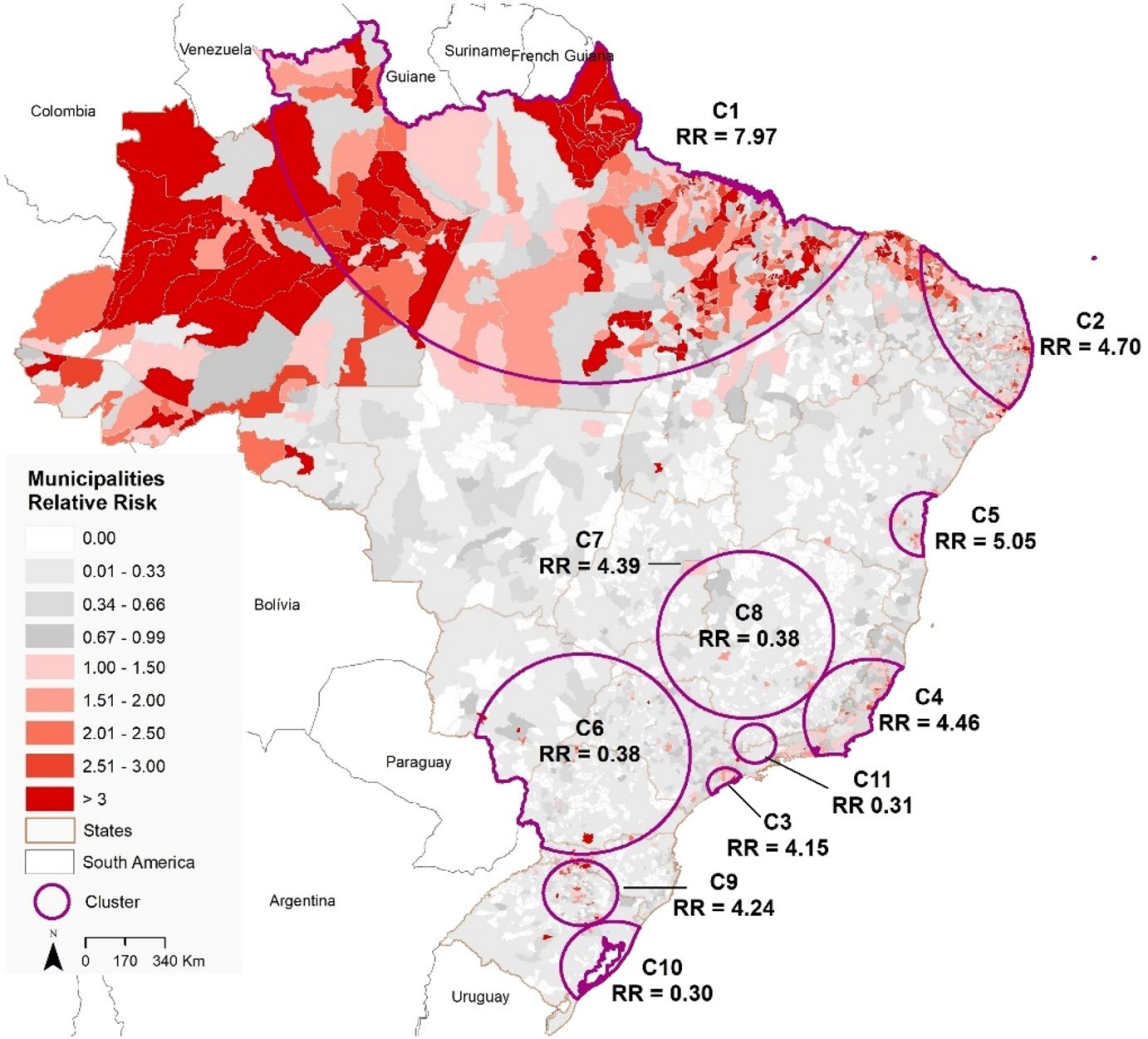
Spatial distribution of emerging space–time clusters of COVID-19 showing the relative risk in Brazil from February 25 to June 7, 2020

We detected 11 emerging space–time clusters of COVID-19 occurring in all Brazilian regions (*p* < 0.001) for the first period (February 25 to June 7, 2020). Among these clusters, three occurred exclusively in the north and northeast regions (Fig. 2). Seven clusters had a relative risk (RR) greater than 1 (i.e., more observed than expected cases). Cluster 1 (RR = 7.97) is located predominantly in the North region and the state of Tocantins (Center-West region) and includes 466 municipalities, with 293 municipalities showing a RR > 1. Cluster 2 (RR = 4.7) is found in the Northeast region and includes 584 municipalities, where 180 have a RR > 1. Cluster 3 (RR = 4.15) is found in the Southeast region, including São Paulo city and 34 municipalities, where 15 have a RR > 1. Cluster 4 (RR = 4.46) includes 274 municipalities and 48 cities with a RR > 1 and is also found in the Southeast region of Brazil, covering the states of Minas Gerais, Espírito Santo and Rio de Janeiro. Cluster 5 (RR = 5.05) includes 68 municipalities in the state of Bahia that is in the Northeast region of Brazil, where eight municipalities have a RR > 1. Cluster 7 includes only Brasília, the Capital of Brazil, with a RR of 4.39 located in Center-West region of Brazil. Finally, Cluster 9 (RR = 4.24) includes 230 municipalities located in Santa Catarina and Rio Grande do Sul states, where 51 municipalities have a RR > 1 (Table 1).

**Table 1:** Emerging space–time clusters of COVID-19 in Brazil from February 25 and June 07, 2020, with relative risk (RR) greater than 1

Cluster	Number of municipalities	Observed	Expected	Cluster RR	Number of municipalities with RR > 1	Time frame*
1	466	142,789	21,929.42	7.97	293	2020/5/5
2	584	94,432	22,556.14	4.7	180	2020/5/4
3	35	81,747	21,633.70	4.15	15	2020/5/5
4	274	50,151	11,909.17	4.46	48	2020/5/18
5	68	12,894	2588.76	5.05	8	2020/5/21
7	1	9791	2252.82	4.39	1	2020/5/15
9	230	6222	1476.71	4.24	51	2020/4/17
Total	1658	398,026	84,346.71	–	596	

*All prospective clusters finish in June 7, 2020

Table 2 shows the first three municipalities with the highest RR for each emerging cluster of COVID-19 identified in Brazil from February 25th to June 7, 2020. We found the highest relative risks in the Amapá state (cluster 1), in the North region of Brazil. The data presented in Table 2 highlighted only three of the Brazilian state capitals (São Paulo and Vitória) and Brasília, calling attention to the highest relative risks in countryside municipalities and some cities along the shoreline.

**Table 2:** Municipalities with the highest relative risk (RR) for each emerging space–time clusters of COVID-19 in Brazil from February 25 and June 07, 2020

Cluster	Region	State	Municipality	Population 2019	Observed cases	Expected cases	RR
1	North	Amapá	Pedra Branca do Amapari	16,502	1166	53.42	21.85
			Serra do Navio	5397	289	17.47	16.54
		Amazonas	Itapiranga	9148	389	29.61	13.14
2	Northeast	Paraíba	Riachão do Bacamarte	4521	118	14.63	8.06
			Caaporã	21,828	480	70.67	6.79
		Ceará	São Gonçalo do Amarante	48,422	993	156.77	6.34
3	Southeast	São Paulo	Santos	433,311	4542	1402.88	3.25
			São Paulo	12,252,023	74,796	39,667.13	1.99
			Guarujá	320,459	1829	1037.51	1.76
4	Southeast	Minas Gerais	Alvarenga	3907	77	12.64	6.08
		Espírito Santo	Vitória	362,097	3508	1172.32	3.00
			Presidente Kennedy	11,574	112	37.47	2.98
5	Northeast	Bahia	Ipiaú	45,873	337	148.51	2.26
			Itajuípe	20,491	145	66.34	2.18
			Uruçuca	20,519	138	66.43	2.07
7	Center-West	Distrito Federal	Brasília	3,015,268	12,864	9762.22	1.32
9	South	Rio Grande do Sul	Lajeado	84,014	1389	272	5.11
		Santa Catarina	Concórdia	74,641	988	241.65	4.09
			Lindóia do Sul	4563	60	14.77	4.06

We observed a critical situation in the Amapá State, where all the municipalities have a RR > 1, and two instances where 50% of the municipalities in two states have a RR > 1 (Table 3). We identified this pattern only in Cluster 1- Pará (62.5%) and Maranhão (62.5%). However, it is important to examine the numbers observed in the Amazonas (Cluster 1 – 45.16%), Paraíba (Cluster 2 – 31.39%), Rio de Janeiro (Cluster 4 – 27.17%), Espírito Santo (Cluster 4 – 25.64%), Ceará (Cluster 2–23.36%) and Roraima (Cluster 1–21.15%) states.

**Table 3:** Number of municipalities with relative risk greater than one (RR > 1) with the percentage based on the number of municipalities for each state that intersects with the emerging space–time clusters (RR > 1) of COVID-19 in Brazil from February 25 to June 7, 2020

Cluster	States intersecting the Cluster	Number of municipalities with RR > 1	% Municipalities with RR > 1 in a state	Number of municipalities in the state
1	Amazonas	28	45.16	62
	Amapá	16	**100**	16
	Maranhão	125	**57.60**	217
	Pará	90	**62.5**	144
	Piauí	5	2.23	224
	Roraima	11	21.15	52
	Tocantins	18	12.94	139
2	Alagoas	16	15.68	102
	Ceará	43	23.36	184
	Paraíba	70	31.39	223
	Pernambuco	35	18.91	185
	Rio Grande do Norte	16	9.58	167
3	São Paulo	15	2.32	645
4	Espírito Santo	20	25.64	78
	Minas Gerais	3	0.35	853
	Rio de Janeiro	25	27.17	92
5	Bahia	8	1.91	417
9	Rio Grande do Sul	38	7.64	497
	Santa Catarina	13	4.40	295

*Cluster 7 is not present in the table, as it contains only the city of Brasília, DF

### Emerging clusters, February 25–July 20, 2020

**Fig. 3:**
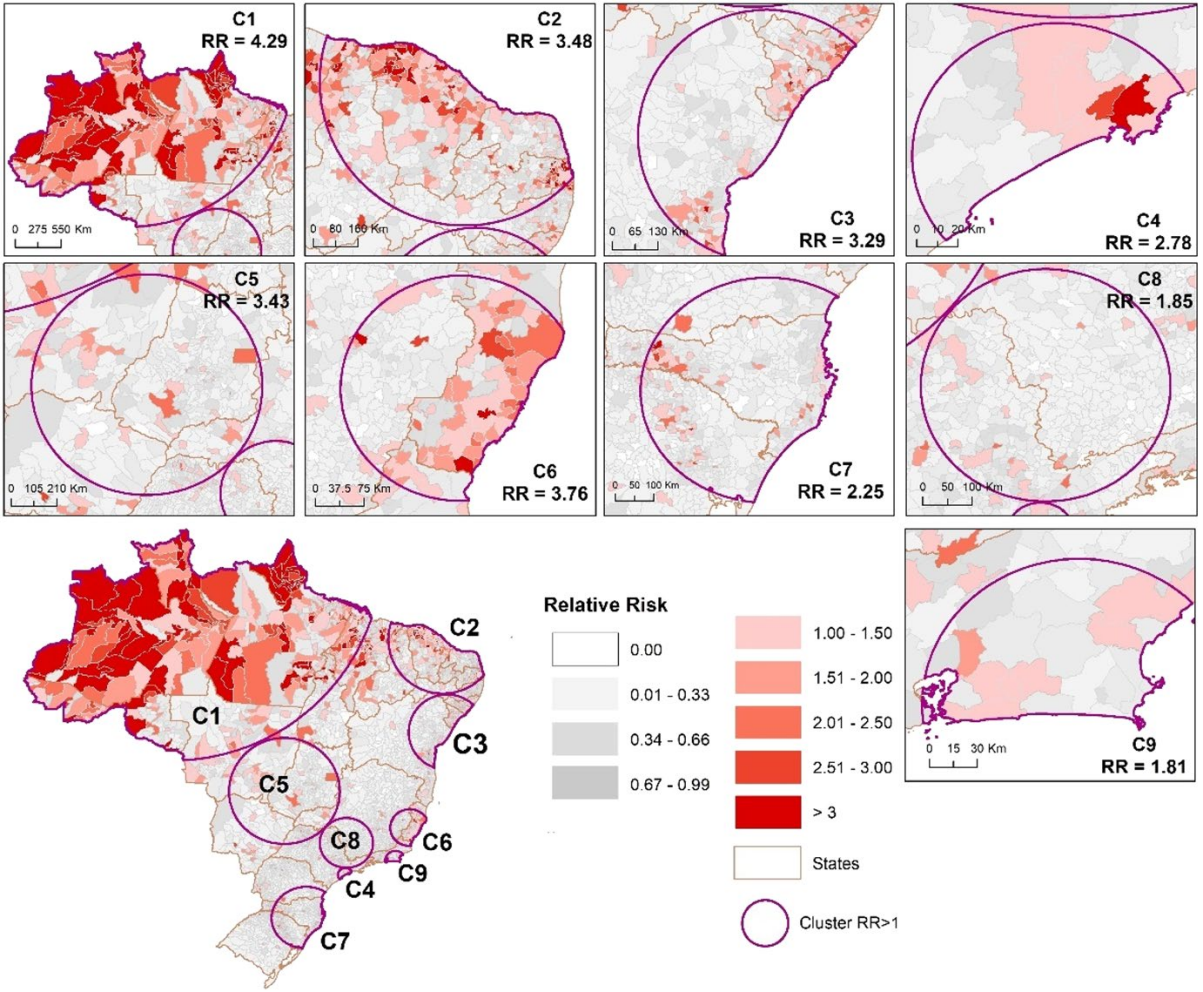
Spatial distribution of emerging space–time clusters of COVID-19 at the municipality level of Brazil from February 25 to July 20, 2020

We detected nine emerging space–time clusters of COVID-19 with a RR > 1, which occurred in all Brazilian regions (*p* < 0.001) for this second period (147 days), while two more clusters were detected compared to the first period of analysis (Fig. 3). In addition, for this time series, the increase in COVID-19 cases in the countryside of Brazil is more evident (Fig. 3). In the first period, we observed 1658 municipalities within an emerging cluster; and in this new time series, we detected 3304 municipalities (an increase of almost 100%). This result shows that almost 60% of the Brazilian municipalities are within an emerging cluster with a RR > 1 (Table 4).

**Table 4:** Emerging space–time clusters of COVID-19 in Brazil from February 25 and July 20, 2020 (RR = relative risk)

Cluster	Number of municipalities	Observed cases	Expected cases	Cluster RR	Number of municipalities with RR > 1	Time frame*
1	513	396,480	207,350.91	4.29	307	2020/5/12
2	755	299,769	207,240	3.48	290	2020/5/28
3	388	191,606	151,849.08	3.29	111	2020/6/2
4	26	244,606	184,980.4	2.78	13	2020/5/27
5	439	163,190	157,465.53	3.43	52	2020/6/15
6	186	88,597	60,006.82	3.76	68	2020/6/2
7	532	112,471	179,440.91	2.25	82	2020/6/23
8	441	116,278	199,931.69	1.86	27	2020/6/19
9	24	98,627	105,658.33	1.81	8	2020/5/18
Total	3304	1,711,624	1,453,923.67	–	958	

*All prospective clusters finish in July 20, 2020

Regarding the number of municipalities with a RR > 1 within the emerging clusters from the first to the second period of analysis, we observed 958 municipalities, an increase of 60.7% (Tables 3 and 4).

Table 5 shows the first three municipalities with the highest RR for each emerging cluster of COVID-19 identified in Brazil from February 25 to June 20, 2020. As observed in the first period of analysis, the municipalities with the highest RR are also located in the northern region of the country. Aracaju is the only state capital that is among the highest RR observed in emerging clusters. Except for Brasília (capital of Brazil), all other municipalities are located in the countryside and some on the shoreline. Only Pedra Branca do Amapari (State of Amapá), Santos (State of São Paulo) Presidente Kennedy (State of Espiríto Santo) and Brasília (Federal district) remained on this list for the two periods analyzed. In this second period, all 26 Brazilian states and Brasília intersect with emerging clusters of COVID-19, while in the first period of the analysis we observed 19 states and Brasília intersecting the with emerging clusters.

**Table 5:** Municipalities with the highest relative risk (RR) for each emerging space–time clusters of COVID-19 in Brazil from February 25 and July 20, 2020

Cluster	Region	State	Municipality	Population 2019	Observed cases	Expected cases	RR
1	North	Pará	Jacareaganga	8239	1195	81.38	14.68
		Amapá	Pedra Branca do Amapari	16,502	2224	162.99	13.64
		Amazonas	Japurá	2755	336	27.21	12.35
2	Northeast	Ceará	Acarape	14,929	803	147.46	5.45
		Paraíba	Guarabira	58,833	2894	581.10	4.98
		Piauí	Demerval Lobão	13,817	653	136.47	4.78
3	Northeast	Sergipe	Aracaju	657,013	24,352	6489.42	3.75
			Cedro de São João	5897	208	58.25	3.57
			Moita Bonita	11,335	352	111.96	3.14
4	Southeast	São Paulo	Santos	433,311	13,340	4279.88	3.13
			Cubatão	130,705	3465	1290.99	2.69
			São Caetano do Sul	161,127	2270	1591.48	1.43
5	Center-West	Distrito Federal	Brasília	3,015,268	70,741	29,782.29	2.42
		Goiás	Rio Verde	235,647	5343	2327.52	2.30
			Palmelo	2381	50	23.52	2.13
6	Southeast	Espírito Santo	Presidente Kennedy	11,574	487	114.32	4.26
			Marechal Floriano	16,694	548	164.89	3.32
		Minas Gerais	Santana do Paraíso	34,663	1036	342.37	3.03
7	South	Rio Grande do Sul	Nova Araça	4759	455	47.01	9.68
		Santa Catarina	Entre Rios	3203	200	31.64	6.32
			Ipuaçu	7514	301	74.22	4.06
8	Southeast	São Paulo	Cordeirópolis	24,528	536	242.27	2.21
			Igaratá	9534	199	94.17	2.11
			Paulínia	109,424	1969	1080.80	1.82
9	Southeast	Rio de Janeiro	Guapimirim	60,517	941	597.74	1.57
			Niterói	513,584	7464	5072.75	1.47
			Macaé	256,672	3574	2535.19	1.41

We observed a critical situation in the Amapá State and Roraima State, where all the municipalities have a RR > 1. In addition to the states of Amapá and Roraima, the states of Amazonas (91.93%), Acre (86.36%), Pará (66.66%), Espírito Santo (62.82%), Ceará (60.32%) and Sergipe (56%) have been in a critical situation with more than 50% of the municipalities with a RR > 1 (Table 6). Some states are intersecting more than one cluster, such as the states of Maranhão (Clusters 1 and 2), Mato Grosso (clusters 1 and 5), Tocantins (clusters and 5), Pernambuco (clusters 2 and 3), São Paulo (clusters 4, 5 and 8), Minas Gerais (clusters 5, 6 and 8) and Rio de Janeiro (clusters 6 and 9).

**Table 6:** Number of municipalities with relative risk higher than one (RR > 1) of COVID-19 in Brazil from February 25 to July 20, 2020

Cluster	States intersecting the cluster	Number of municipalities with RR > 1	% Municipalities with RR > 1 in a state	Number of municipalities in the state
1	Acre	19	86.36	22
	Amazonas	57	91.93	62
	Amapá	16	100	16
	Maranhão*	57	26.26	217
	Mato Grosso*	14	9.92	141
	Pará	96	66.66	144
	Rondônia	11	21.15	52
	Roraima	15	100	15
	Tocantins*	37	26.61	139
2	Ceará	111	60.32	184
	Maranhão*	9	4.14	217
	Paraíba	80	35.87	223
	Pernambuco*	7	3.78	185
	Piauí	45	20.08	224
	Rio Grande do Norte	38	22.75	167
3	Alagoas	37	36.27	102
	Bahia	31	7.43	417
	Pernambuco*	1	0.54	185
	Sergipe	42	56	75
4	São Paulo*	13	2.01	645
5	Distrito Federal	1	100	1
	Goiás	15	6.09	246
	Minas Gerais*	6	0.70	853
	Mato Grosso do Sul	2	2.53	79
	Mato Grosso*	8	5.67	141
	São Paulo*	19	2.94	645
	Tocantins*	1	0.71	139
6	Espírito Santo	49	62.82	78
	Minas Gerais*	12	1.40	853
	Rio de Janeiro*	7	7.60	92
7	Paraná	1	0.25	399
	Rio Grande do Sul	33	6.63	497
	Santa Catarina	48	16.27	295
8	Minas Gerais*	5	0.58	853
	São Paulo*	22	3.41	645
9	Rio de Janeiro*	8	8.69	92

*States that intersected more than one cluster

### Regression results

**Fig. 4:**
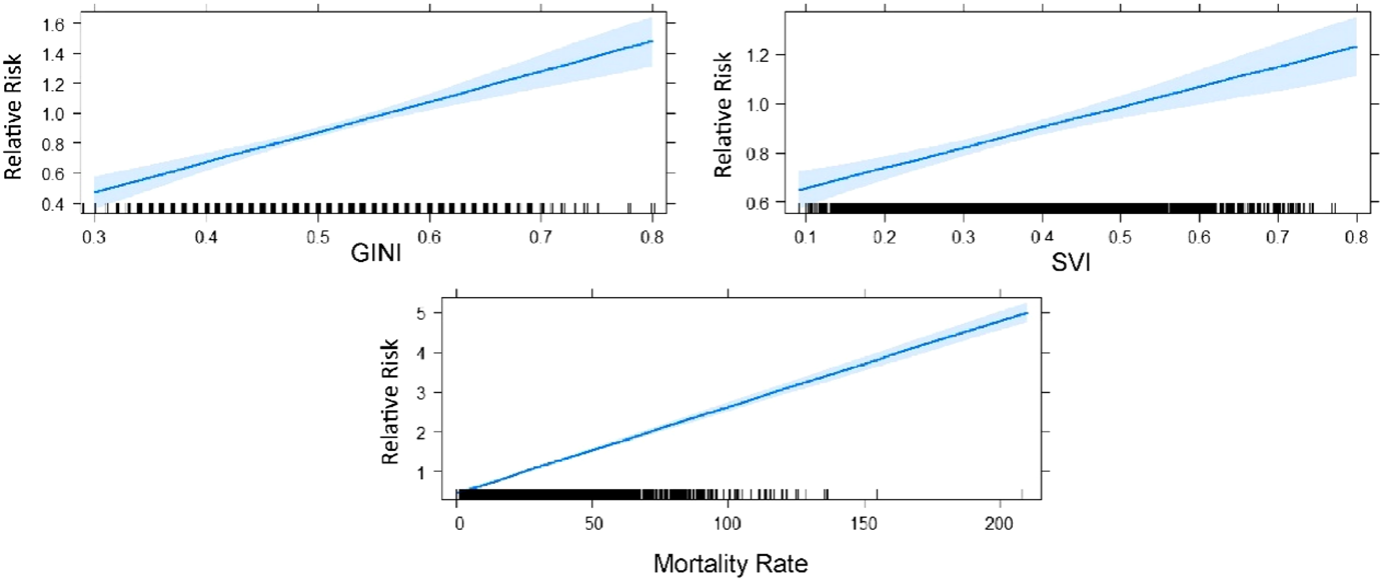
Linear fit of the Generalized Linear Models (blue line) with confidence intervals (shaded area) for relative risk of the municipalities belonging to the space–time clusters of COVID-19 in Brazil

The results obtained through the GLM showed that there was a significant positive correlation between the predictor variables in relation to the relative risk at the level of the municipalities belonging to the nine emerging clusters (Fig. 4).

The GLM result showed that all variables have a statistically impact on the RR of the municipalities located in the clusters. For each one-unit increase in the GINI variable, the relative risk increases by 2.02, while the impact of SVI is 0.82, and mortality rate is 0.021. For comparison purposes, we implemented the same model for all municipalities in Brazil regardless of their cluster membership, and all municipalities outside the clusters. When applied to all municipalities, the same predictor variables were significant; however, the estimates were substantially lower (closer to zero) with a decrease in the standard error. The decrease in the estimate was even more pronounced when the model was conducted to the municipalities outside of the clusters.

We identified problems of multicollinearity and heteroscedasticity (Appendix Table 13), in addition to spatial dependence on residuals, confirmed by the Moran’s I statistic (Appendix Fig. 5). Although the Moran’s I test showed a value close to zero (Table 7), it is still possible to identify spillovers with groups of high–high in the Brazil North region and others of low–low, mainly in the Northeast region. Table 8 also shows the result of the Lagrange Multiplier Test, which indicated the recommendation for applying spatial regression for LM-Lag and LM-Error regressions.

**Fig. 5:**
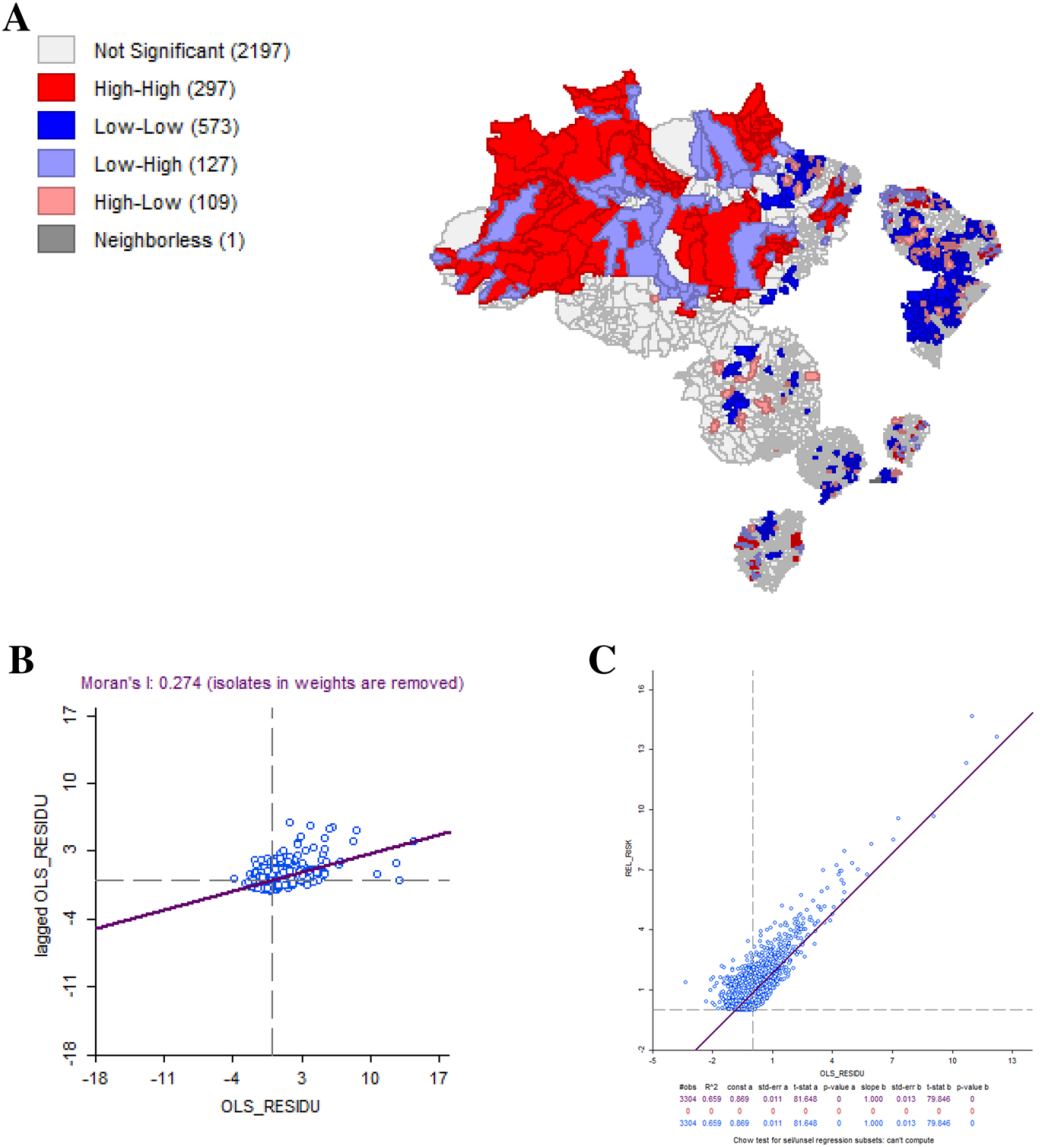
a Lisa map for GLM residual, **b** Moran’s I scatterplot residual, **c** residual scatterplot for GLM

**Table 7:** Estimated parameters of the predictor variables used for the Generalized Linear Model for the relative risk of COVID-19 for the municipalities belonging to the space–time clusters (R^2^ = 0.34)

Variable	Coefficient	SE	*p*
Constant	0.8686	0.0148	< .001
GINI	2.0204	0.2718	< .001
SVI	0.8208	0.1350	< .001
Mortality rate	0.0217	0.0006	< .001

Akaike info criterion: 8306.26, R^2^: 0.341210

**Table 8:** Spatial dependence diagnostic

Test	MI/DF	Value	PROB
Moran’s I (error)	0.3426	32.0821	0.000
Lagrange multiplier (lag)	1	1173.7476	0.000
Robust LM (lag)	1	174.2724	0.000
Lagrange multiplier (error)	1	1020.7702	0.000
Robust LM (error)	1	21.2950	0.000
Lagrange multiplier (SARMA)	2	1195.0426	0.000

Considering both spatial regressions performed, LM-Lag showed the most adjusted model based on the AIC values (Appendix Figs. 6, 7), as well as in comparison to the GLM (Appendix Table 14). The result of the Moran’s I test LM-Lag and LM-Error residuals was 0.274 and -0.033, respectively. Based on these results, we choose the LM-Lag spatial regression model, which is more adjusted to assess how the variables GINI, SVI and Mortality Rate explain the variation in the RR of the municipalities located within the clusters. The results of the LM-Lag showed that all variables analyzed have a statistically significant effect for RR, except for the SVI (Table 9).

**Fig. 6:**
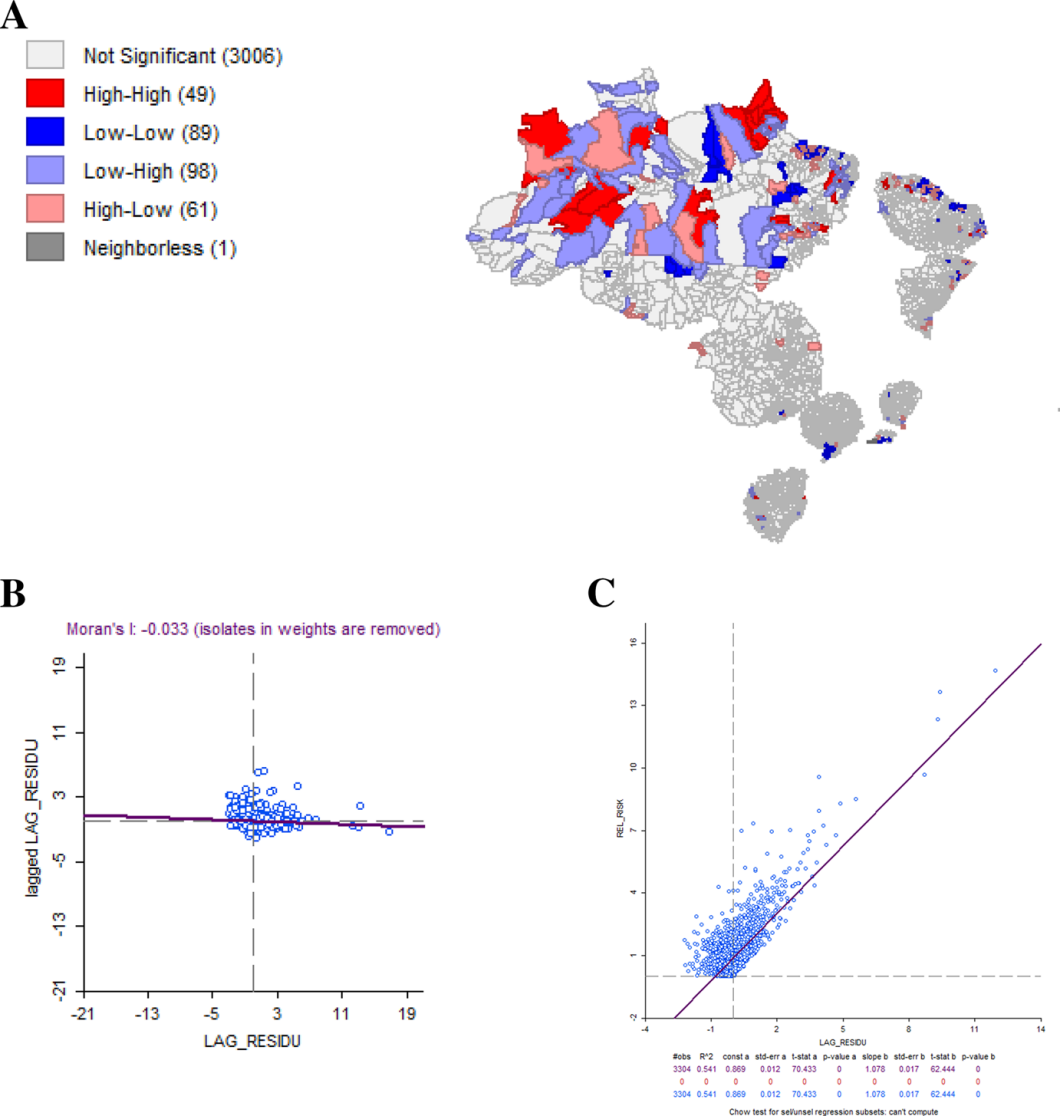
a Lisa map for LM-lag residual, **b** Moran’s I scatterplot residual, **c** residual scatterplot for LM-Lag

**Fig. 7:**
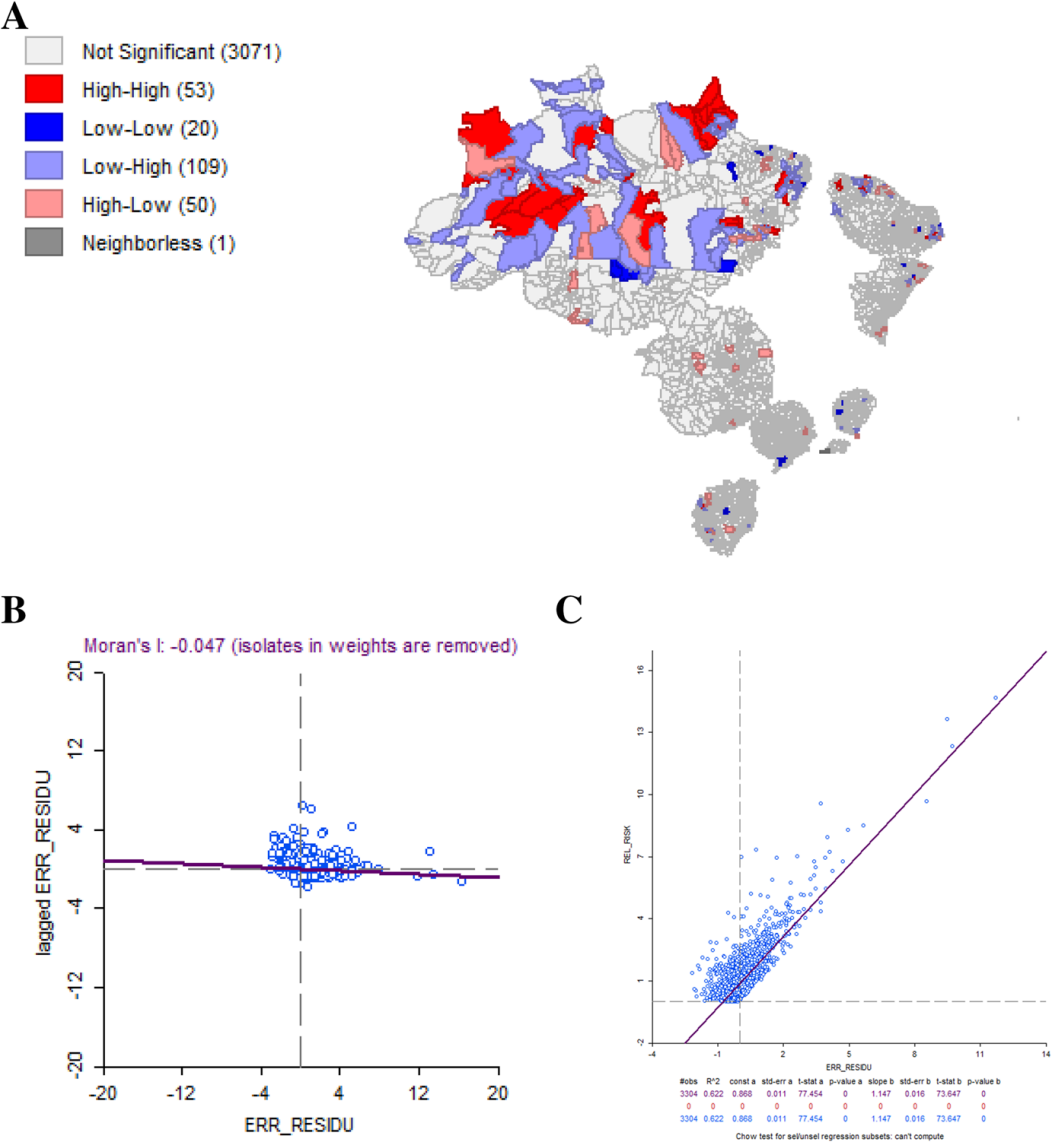
a Lisa map for LM-error residual, **b** Moran’s I scatterplot residual, **c** residual scatterplot for LM-Error

**Table 9:** Estimated parameters of the variables used for the LM-Lag for the relative risk of COVID-19 for the municipalities belonging to the space–time clusters

Variable	Coefficient	SE	z-value	Probability
W_Relative Risk	0.55969	0.016697	33.52	0.00000***
CONSTANT	− 0.44866	0.095822	468.226	0.00000***
GINI	118.502	0.229002	517.471	0.00000***
SVI	− 0.08561	0.11544	0.74159	0.45834
Mortality Rate	0.014232	0.000597	238.302	0.00000***

Signif. codes: 0 ‘***’ 0.001 ‘**’ 0.01 ‘*’ 0.05 ‘.’ 0.1 ‘’

According to the results of the model, the variables GINI and mortality rate have a significant effect (*p* < 0.001) on the relative risk of municipalities within the clusters. Table 10 shows the results about the direct, indirect, and total effects. The GINI variable has the greatest positive direct (1.280) and indirect (1.414) effects, resulting in a total effect of 2.695. This indicates an increase in the RR within the municipalities due to inequality, but mainly due to the role of spillovers from neighboring municipalities. The same pattern was also observed for the mortality rate for the direct (0.015), indirect (0.016), and total (0.032) effects. In the case of SVI, no significant effects were observed for RR (Table 10).

**Table 10:** Direct, indirect, and total effects of the model

Dependent variables	Direct	Indirect	Total
SVI	− 0.09314893	− 0.10293739	− 0.19608632
Simulated p-values	0.20196	0.20293	0.20228
GINI	1.2802934	1.41483177	2.69512519
Simulated p-values	<0.001***	<0.001***	<0.001***
Motality rate	0.01534331	0.01695565	0.03229895
Simulated p-values	<0.001***	<0.001***	<0.001***

Signif. codes: 0 ‘***’ 0.001 ‘**’ 0.01 ‘*’ 0.05 ‘.’ 0.1 ‘’

### Sensitivity of scan parameters

In this paper, we selected spatial and temporal scan statistic parameters in line with previous work (Desjardins et al. 2020; Hohl et al. 2020a, b). We conducted a sensitivity analysis by parameterizing different spatial and temporal scanning windows: (1) 25% of the population at-risk and 25% of the study period; (3) 10% and 50%, (3) 10% and 25%, (4) 5% and 50%, (5) 5% and 25%, and (6) 5% and 10%. In general, increasing the temporal scan results in clusters that start much earlier than smaller search windows (e.g., 10% versus 50%), essentially capturing a longer temporal range of cases. As such, increasing the temporal cutoff too much may obscure smaller space–time clusters that happened more recently in Brazil. Spatially, the general location of the clusters is very similar when decreasing the spatial scanning window from 25% to 5%. Larger spatial scanning windows may encompass multiple clusters that were detected using smaller windows (e.g., 25% versus 5%). This is due to the relaxation of the population constraint, allowing the windows to capture more municipalities which result in very large clusters in rural regions of Brazil (e.g., State of Amazonas). The spatial overlap of the clusters using different scanning windows provide some confidence that the COVID-19 clusters are “stable”, despite the parameter selection.

## Discussion

Comparing the first period (103 days) with the second (with an increase of 44 days), the results show a significant increase in the COVID-19 in Brazil, demonstrated by the 61% increase in the number of municipalities with RR > 1 within the detected clusters. A significant increase in the number of municipalities was detected, confirming the study by Peixoto et al. (2020) who showed that not only risk regions are those closest to the capitals where the outbreak began, but that there are also interior cities with risk.

The choropleth maps presented in this paper (Figs. 2 and 3) are based on the calculations of the relative risk, which reflects the relationship between the total number of confirmed cases in relation to expected cases based estimated from the population of the municipality. In the first time period (February 25–June 7), municipalities with RR > 1 where we identified mainly in the North, Northeast and close to the coast associated with the occurrence of the main state capitals, such as São Paulo and Rio de Janeiro. However, in the second period (February 25 to July 20), there was an increase in the number of municipalities with RR > 1 in the North and also in the coastal regions. Besides that, there was also an increase in the number of municipalities with RR > 1 in the interior of the country. Costa et al. (2020) identified that some states had outbreaks that started mainly in their capitals, followed by epidemic waves that spread toward the interior, and that still other states have multiple initial outbreaks of epidemics.

The inequality (GINI) and mortality rate have direct, indirect, and total positive effects of relative risk detected in the Brazilian municipalities. This finding is related to the spread of COVID-19 to the countryside, where there is high social inequality, mainly in the municipalities of the North and Northeast Regions (74.3% of the municipalities with RR > 1). The states of the South and Southeast regions have a lower concentration of income, while the Center-West, North, and Northeast regions have higher levels of inequality (Colombo and Ferreira 2019). Coelho et al. (2020) highlighted that these areas would suffer an increased spread of the disease in populations with greater socioeconomic vulnerability.

When bringing to debate the political aspect of the virus, Smith and Judd (2020) consider relevant “to reflect on who is most vulnerable in pandemics”. This question is based on the argument that, despite the fact that COVID-19 can affect the whole of society, its effects will be experienced in different ways, depending on the level of equity that exists in each social reality, as such it is essential to analyze the pandemic and the policies that emanate from it in the perspective, not only of health, but also of social and economic determinants (Smith and Judd 2020).

The municipalities with the greatest inequality will likely be the regions with the highest incidence and death of COVID-19 in Brazil. Our results indicate that inequality is a significant variable that explains RR increase in the municipalities with spatial spillover effects. The inequality of income and the lack of access to services are sufficient to suggest that there is a disproportionate effect of COVID-19 among the most vulnerable in the country (Pires et al. 2020). In addition, there is a lack of protocols and measures aimed at the social protection of these populations in the atypical context of a pandemic; so when clusters with high risk show a high mortality rate, this can guide decisions for these municipalities.

The relationship of active clusters with indices that express inequality in the country may represent the beginning of a problematic scenario, especially so in the most vulnerable municipalities. This may result from the difficulties of enforcing social isolation due to the needs of maintaining employment and income, as well as access to health and basic sanitation (Pires et al. 2020). In the study carried out by Fiocruz (2020) on April 2nd, 2020, the most vulnerable regions of Brazil were identified in the north and northeast. In our study, we found the highest relative risk in north and Northeast for all-time series, corroborating with Fiocruz (2020). Although the North Region of Brazil has low levels of urbanization, river migration is related to the spread and progression of the disease in the municipalities of the State of Amazonas, unlike other urbanized regions (Aleixo et al. 2020).

Examining the adherence to social distancing guidelines requires a more detailed analysis, which was beyond the scope of this study. Amazonian communities such as Indians and riverside populations are geographically isolated populations; however, they have been impacted by COVID-19 (ISA 2020). Therefore, actions need to be taken based on the geographic, social, and cultural differences than those living in urban areas. We present an exploratory study that identifies associations between the relative risk of COVID-19 clusters and mortality, inequality, socioeconomic vulnerability of the disease in Brazilian municipalities, but there are not enough elements to detail demographic particularities of the population.

An important correlation between clusters with high relative risk and mortality rates and inequality is observed, but the method is sensitive to the scale adopted (Chen et al. 2008); therefore, we hypothesize that the findings will be different and may be more severe regarding relative risk if finer-level data were available. The pandemic is still spreading in Brazil, and it is difficult to estimate the speed of transmission along the countryside, where small population municipalities are located. However, our research highlights the regions that are experiencing the highest risk of COVID-19, which is critical for improving public health decision-making. Preventive measures must be strengthened and adhered to, while the only strategy that has proved effective for the control of COVID-19 has been social distancing (de Oliveira et al. 2020).

Despite the strengths of this study, there are several of limitations worth addressing in future studies. First, the cylindrical scanning windows of the prospective scan statistic may not capture the true shape of the COVID-19 outbreaks. However, this is an exploratory study and cylindrical scanning windows are widely used and acceptable in the SaTScan literature. We encourage more research on developing irregular search windows, similar to by Tango and Takahashi (2005) and Wu and Grubesic (2010), but extended in time. Second, we do not provide daily surveillance of COVID-19 in Brazil, as this was beyond the scope of our research objectives. Rather, we provide two snapshots of the situation in South America’s largest country. Future research can provide daily updates, similar to Hohl et al. (2020b). Third, there are inherent biases in the dataset that we used. Like many other countries, testing was not always accessible when the pandemic reached Brazil; as such findings may have suffered from this underreporting, although the country ramped up testing. Fourth, we used different covariates to explain the variation in relative risk (inside and outside clusters). One avenue worth modeling as future research is the persistence of a cluster over time, an approach suggested by He et al. (2017). This approach is worth pursuing when the timespan of study would be long enough. Finally, we did not adjust the p-values for multiple analyses; however, Kulldorff and Kleinman (2015) suggest that adjusting p-values should be avoided for long sequences of data, especially in a spatiotemporal context (i.e., increasingly difficult to reject null hypothesis).

## Conclusion

This research presented an analysis of the dynamics of the expansion of COVID-19 based on the number of daily cases by municipality, with the intent of identifying emerging space–time clusters active in Brazil in the first five months of the pandemic. We detected nine significant active clusters of COVID-19 within Brazil on July 20, 2020. Therefore, this space–time approach to detect emerging clusters will allow decision-makers to identify statistically significant hotspots of COVID-19 cases. States are responsible for coordinating the activities at the regional health level. These regions can use these results to optimize coordination and organization of health care needs, specifically in relation to the poorest populations and those with the highest health-care demand. Our approach may also allow authorities to pay attention to municipalities that still have little-to-no cases, so they can be prepared to face the burdens of COVID-19. In turn, this can improve the management of resources to the States and Health Regions.

## Appendix

See Tables 11, 12, 13, 14, 15, 16, 17, 18 and Figs. 5, 6, and 7.

**Table 11:** Variables, sources, units and links to access the data used in this research

Variable	Source	Units	URL
COVID-19 cases and deaths	Brazil in released data (Brasil.io), 2020	Count	https://brasil.io/covid19/
Municipality polygons	Brazilian Institute of Geography and Statistics (IBGE), 2010	Polygonal geometry	https://www.ibge.gov.br/geociencias/organizacao-do-territorio/malhas-territoriais.html
Municipality city points	Brazilian Institute of Geography and Statistics (IBGE), 2010	Points in lat/long coordinates	https://geoservicos.ibge.gov.br/geoserver
Population estimation	Brazilian Institute of Geography and Statistics (IBGE), 2019	Count	https://www.ibge.gov.br/estatisticas/sociais/populacao/9103-estimativas-de-populacao.html?edicao=25272&t=resultados
GINI coefficient	Atlas of Human Development in Brazil, 2010	Coefficient	http://atlasbrasil.org.br/2013/pt/consulta/
Social Vulnerability Index (SVI)	Atlas of Social Vulnerability, 2013	Coefficient	http://ivs.ipea.gov.br/index.php/pt/planilha

**Table 12:** Descriptive statistics for the variables used in the GLM model

	GINI	SVI	Mortality rate	Observed cases
Mean	0.494	0.352	15.7	373
Median	0.49	0.335	7.42	46
Sum	2754	1958	87724	2075657
Standard deviation	0.0661	0.13	21.9	2974
Variance	0.00437	0.0169	481	8,840,000
Minimum	0.28	0.09	0	0
Maximum	0.8	0.784	208	166,348
25th percentile	0.45	0.246	0	12
50th percentile	0.49	0.335	7.42	46
75th percentile	0.54	0.448	23	180

**Table 13:** GLM regression diagnostics

Test	DF	Value	Prob.
Multicollinearity: test on normality of errors			
Jarque–Bera	2	233,139.5728	0.00
Heteroskedasticity (random coefficients)			
Breusch–Pagan test	3	2644.6462	0.00
Koenker–Bassett test	3	125.1991	0.00

**Table 14:** Spatial lag model coefficients

Variable	Coefficient	SE	z-value	Probability
W Relative risk	0.55969	0.016697	33.52	0.00000***
Constant	− 0.44866	0.095822	− 468.226	0.00000***
GINI	118.502	0.229002	517.471	0.00000***
SVI	− 0.08561	0.11544	− 0.74159	0.45834
Mortality rate	0.014232	0.000597	238.302	0.00000***

Signif. codes: 0 ‘***’ 0.001 ‘**’ 0.01 ‘*’ 0.05 ‘.’ 0.1 ‘’

Akaike info criterion: 7385.64

**Table 15:** Spatial lag model regression diagnostics

Test	DF	Value	Prob.
Heteroskedasticity (random coefficients)			
Breusch-Pagan test	3	3092.3383	0.00
Spatial dependence			
Likelihood ratio test	1	922.6248	0.00

**Table 16:** Spatial error model regression coefficients

Variable	Coefficient	SE	z-value	Probability
Constant	− 0.0613326	0.127557	− 0.480826	0.63064
GINI	118.985	0.249604	476.696	0.000***
SVI	0.120072	0.172886	0.694515	0.48736
Mortality rate	0.0160655	0.000671939	239.091	0.000***
Lambda	0.617606	0.0177266	348.406	0.000***

Signif. codes: 0 ‘***’ 0.001 ‘**’ 0.01 ‘*’ 0.05 ‘.’ 0.1 ‘’

Akaike info criterion: 7490.79

**Table 17:** Spatial error model regression diagnostics

Test	DF	Value	Prob.
Heteroskedasticity (random coefficients)			
Breusch-Pagan test	3	2995.6530	0.00
Spatial Dependence			
Likelihood Ratio Test	1	815.4676	0.00

**Table 18:** Result of the Akaike information criterion (AIC) for the regression models

Regression models	AIC
Generalized Linear Model (GLM)	8306.26
Lagrange Multiplier test for spatial lag dependence (LM-lag)	7385.64
Lagrange Multiplier test for spatial error dependence (LM-Error)	7490.79
